# Beyond the Guidelines: Perspectives on Management of Pediatric Patients with Hypertriglyceridemia

**DOI:** 10.1007/s11883-024-01237-z

**Published:** 2024-09-30

**Authors:** Charles A. Gagnon, Ambika P. Ashraf

**Affiliations:** 1 University of Alabama at Birmingham Marnix E. Heersink School of Medicine, Birmingham, AL USA; 2 Division of Pediatric Endocrinology and Diabetes, Department of Pediatrics, University of Alabama at Birmingham, Birmingham, AL USA

**Keywords:** Hypertriglyceridemia, Hyperchylomicronemia, Fasting Triglyceride, Triglyceride Rich Remnant Lipoprotein, Lipoprotein Lipase, Apo CIII, Microsomal Triglyceride Transfer Protein, ANGPTL3

## Abstract

**Purpose of Review:**

To provide a comprehensive overview of hypertriglyceridemia (HTG) in youth, identifying gaps in categorizing triglyceride (TG) levels and management strategies, and exploring new therapies for TG reduction.

**Recent Findings:**

Non-fasting TG levels as important cardiovascular (CV) risk indicators, with HTG's pathophysiology involving genetic and secondary factors affecting TG metabolism. Emerging treatments, including those affecting the lipoprotein lipase complex and inhibiting proteins like apoC3 and ANGPTL3, show promise.

**Summary:**

The review highlights the need for specific management approaches for youth, the significance of non-fasting TG levels, and the potential of new therapies in reducing CV and pancreatitis risks, advocating for further research on these treatments' efficacy and safety.

## Introduction

Hypertriglyceridemia (HTG) is generally defined as a fasting plasma triglyceride (TG) levels exceeding the 95th percentile for age and sex [1]. This equates to a TG concentration of ≥ 100 mg/dL for children ages 0–9 years of age and ≥ 130 mg/dL for those aged 10–19 [2]. According to National Health and Nutrition Examination Surveys (NHANES) conducted from 1999 to 2006, HTG prevalence increased with body mass index (BMI), reaching 10.2% in adolescents, 13.8% in overweight children, and 24.1% in children with obesity [3]. Other studies suggest an even higher prevalence, ranging from 30 to 60% among overweight or obese youth [4–6].

HTG involves a variety of TG-rich lipoproteins (TGRLP), such as very low density lipoprotein (VLDL) from the liver, chylomicrons from the intestine, and their intermediaries, like intermediate density lipoprotein (IDL), and chylomicron remnants [7]. Chylomicrons and IDL are usually cleared from the plasma through hydrolysis of TG by lipoprotein lipase (LPL). Within a few hours after a meal, these are mostly cleared out of the bloodstream. Consequently, fasting plasma TG levels primarily reflect circulating VLDL.

Clinically, mild to moderate HTG, caused by elevated VLDL concentration, is associated with a higher risk of premature cardiovascular disease (CVD). Severe HTG, due to accumulation of chylomicrons, is linked to pancreatitis risk. The risk of acute pancreatitis increases with serum TG levels exceeding 500 mg/dL (5.6 mmol/L), reaching 5% at 1000 mg/dL (11.3 mmol/L), and 10–20% over 2000 mg/dL (22.6 mmol/L) [8, 9].

Management of HTG is complex due to its heterogeneous nature. The article aims to synthesize existing data on HTG, offering an overview of current and emerging management approaches, while identifying gaps and opportunities for future research.

## Definitions

A classification of TG levels presents challenges due to varying guidelines by professional societies over the years [10–13]- See Table 1. The definitions of mild, moderate, and severe HTG differ significantly among professional societies with thresholds ranging from 150 mg/dL to over 2000 mg/dL. This lack of consensus makes it challenging to consistently categorize HTG in the clinical setting.

**Table 1:** Classifications of Hypertriglyceridemia (HTG)

Professional Society	Mild HTG (mg/dL)	Moderate HTG (mg/dL)	High TG (mg/dL)	Severe TG (mg/dL)	Very Severe HTG (mg/dL)
The National Cholesterol Education Program Adult Treatment Panel III (2001) National Lipid Association (2015)	150–199		200–499	≥ 500	
American College of Cardiology/ American Heart Association (2019)		150–499		≥ 500	
Endocrine Society (2012)	150–199	200–999		1000–1999	≥ 2000

The National Cholesterol Education Program Adult Treatment Panel III (2001) and the National Lipid Association (2015) guidelines define mild HTG as 150-199 mg/dL, high HTG as 200-499 mg/dL, and very high HTG as over 500 mg/dL [10, 11]. The 2019 American College of Cardiology (ACC)/ American Heart Association (AHA) define moderate HTG as 150–499 mg/dL and severe HTG as 500 mg/dL or higher [12]. The Endocrine Society (2012) guidelines classify mild HTG as 150–199 mg/dL, moderate HTG as 200-999 mg/dL, severe HTG as 1000–1999 mg/dL, and very severe HTG as 2000 mg/dL or higher [13]

*HTG* Hypertriglyceridemia

From a management perspective the National Heart, Lung, and Blood Institute (NHLBI) recommends pharmacotherapy to prevent pancreatitis in youth < 18 years of age when TG levels exceed 500 mg/dL [2]. NHLBI recommends considering non-HDL cholesterol (non-HDL-C) when the TG levels are below 500 mg/dL. Non-HDL cholesterol can be calculated (Total cholesterol - HDL-C) using a fasting or nonfasting sample [2, 14, 15]. Calculating non-HDL-C is crucial, especially when it differs from LDL-C as seen in combined dyslipidemia. This discrepancy can indicate the presence of additional atherogenic particles, such as VLDL-C, suggesting a higher residual burden of apoB-containing particles.

The 2019 AHA guidelines in high-risk pediatric patients classify TG as moderate, significant and severe risk [16]: The classifications are as follows:

- **Moderate risk:** TG levels below 400 mg/dL and non-HDL-C more than 145 mg/dL; treatment may involve statins based on the risk category.
- **Significant risk:** TG between 400–999 mg/dL.
- **Severe risk:** TG levels exceeding 1000 mg/dL.

### Triglyceride Metabolism

Understanding the metabolic pathway of triglycerides is crucial, as it significantly influences how we approach HTG.

#### Chylomicron production

Dietary fat primarily exists as triacylglycerol (TAG), which undergoes hydrolysis by the intestinal lipases into free fatty acids (FFA) and monoglycerides. These products are emulsified by bile salts and phospholipids, forming micelles that are absorbed into enterocytes. Within the intestinal cells, FFA enters enterocytes and combine to form TG. The resulting TG (85%) and cholesteryl ester (CE) are assembled in the gut and packaged into chylomicrons, along with apo B48. When fat is absorbed in the human body, it enters the circulation as intestinally derived TG-rich lipoproteins, primarily in the form of chylomicrons, typically within about 15 min after finishing a meal [17].

Intravascular TGs undergo catabolism into FFA through the action of lipoprotein lipase (LPL) in the capillary lumen, liberating FFA for energy use. Thus, chylomicron metabolism bypasses the liver. If LPL function is impaired or absent, the triglyceride within the circulating chylomicron are not broken down into FFA for cellular  energy use, leading to elevated circulating chylomicron triglyceride levels that remain in the bloodstream. After lipolysis, the chylomicron remnant which contains CE, retinyl esters, and apoB-48, enters the liver and the TG and cholesterol content becomes incorporated into VLDL. ApoC-II serves as the primary cofactor which facilitates LPL actions, while apoC-III inhibits LPL function and prevents the uptake of lipoproteins by liver receptors. ApoE acts as the ligand, facilitating hepatic uptake of TG-rich remnant lipoproteins.

#### VLDL Synthesis

As mentioned above, hepatocytes play a key role in synthesizing VLDL, which is secreted into plasma. An excess intake of dietary carbohydrates leads to de novo FA synthesis and elevated FFA levels. Insulin resistance (IR) also increases the release of FFA from adipocytes, stimulating the liver to produce apo B100 and, consequently, TG-rich VLDL.

In the circulation, cholesteryl ester transfer protein (CETP) facilitates the exchange of CE from HDL to LDL and VLDL, enriching these apo B containing lipoproteins with cholesterol. Conversely, CETP mediates the transfer of TG from LDL and VLDL to HDL, resulting in TG-enriched HDL. This process reduces the size of VLDL and transforms it into IDL, which can then be hydrolyzed by hepatic lipase (HL) to form LDL. TG-enriched LDL undergoes lipolysis, becoming small, dense, LDL (sdLDL) particles that are atherogenic. During this process, apoA-I dissociates from TG-enriched HDL and is cleared from plasma through the kidney. Patients with HTG often have lower levels of HDL.

### Current Debates in the Field

#### Fasting and TG Measurement

Ideally, TG concentration is measured after a 10–12 h fast. However, use of non-fasting samples are becoming more common. Despite a expected rise in TG post-prandially, other lipid measurements, such as total cholesterol, low density lipoprotein cholesterol (LDL-C), and HDL-C, change minimally after an average meal [18]. TG typically increases by 17.7 to 35.4 mg/dL (0.2 to 0.4 mmol/L) two to six hours after a meal [19–21]. In patients with impaired TG metabolism, TG concentrations can increase by up to four times following a meal, depending on the fat content of the meal and LPL complex functionality [18]. In most cases, a non-fasting lipid level can be assessed initially. If the TG level is elevated, a fasting lipid measurement can be undertaken.

*Importance of Non-Fasting TG Levels and CV Health:* Recent evidence suggests that non-fasting TG levels may impact CVD risk [22]. Two cohort studies, the Women’s Health Study in the United States and the Circulatory Risk in Communities Study in Japan, highlight the predictive power of non-fasting TG levels for CVD events [23, 24]. Similarly, the Copenhagen City Heart Study found a link between elevated non-fasting TG levels and atherosclerotic cardiovascular disease (ASCVD) events, as well as all-cause mortality [25, 26]. There have been no studies in children that have demonstrated an association between non-fasting TG and CV health.

*Remnant Lipoproteins in Non-Fasting TG:* Levels of non-fasting TG reflect increased TG rich lipoprotein (TRL) remnants [25], and are atherogenic since they contain substantial amounts of cholesterol and apoB100, prone to entrapment within the arterial wall [27]. In contrast, chylomicrons, are too large to penetrate the arterial intima and contain apoB-48 as their apolipoprotein [28, 29].

*Lack of Standardization of Non-Fasting TG Measurement:* Non-fasting TG offers convenience with a single blood draw. Several organizations, including the AHA, European Atherosclerosis Society, and Danish Society for Clinical Chemistry, advocate for non-fasting lipid panels in adults [30–32]. European and American Heart Association guidelines define ideal non-fasting TG levels as < 175 mg/dL and < 200 mg/dL, respectively [30, 31]. Optimal levels for non-fasting TG have not been established in children.

*CV Risk and TG Lowering:* The efficacy of reducing TGs in decreasing ASCVD risk continues to be debated. While the association between TG and CV is evident in observational studies [25, 33–36]; randomized controlled trials (RCTs) targeting TG reduction have yielded overall positive but inconsistent results regarding major vascular events [37].

It has been suggested, however, that a 89 mg/dL increase in non-fasting TG is associated with 2.8-fold increase in CVD risk [38]. One of the difficulties of defining  role of TG in cardiovascular disease risk is that elevated levels are generally associated with an atherogenic environment, often involving sdLDL and diminished HDL-C levels. SdLDL particles exhibit enhanced endothelial penetration, retention, and susceptibility to oxidation [39] – all promoting atherogenesis. Both HTG and metabolic syndrome are associated with a hypercoagulable state, further increasing CVD risk [40]. Thus, HTG likely accelerates atherosclerosis through multiple mechanisms, raising CVD risk.

## Etiology

Hypertriglyceridemia may occur from increased production, impaired clearance, or a combination of both—Table 2. With levels < 1000 mg/dL, elevated TG mainly reflect increased VLDL synthesis in the liver. At higher TG concentrations, reduced TG clearance due to dysfunction in the LPL complex plays a significant role in elevating TG, leading to predominantly buildup of chylomicron.

**Table 2:** Etiology and Lipid Phenotype of Hypertriglyceridemia

Disorder	Etiology	Defect	Lipid phenotype	Triggers
Mild to Moderate (< 1000 mg/dL)				
FCHL	AD with variable penetrance	Apo B100 overproduction	Combined hyperlipidemia TG < 500 mg/dL (5.6 mmol/L)	Can progress to severe HTG in the presence of secondary factors
Familial HTG	AD	Enlarged VLDL	TG < 500 mg/dL, (5.6 mmol/L) normal LDL-C and HDL-C levels	Can progress to severe HTG in the presence of secondary factors
Dysbeta lipoproteinemia	Complex, AR	*APOE* mutation (*E2/E2* genotype)	Equal elevation of TG and cholesterol (remnant particle)	In conjunction with FCHL or with secondary causes
Familial hypoalphalipoproteinemia (FHA)	Tangiers-AD LCAT deficiency: AR		Elevated TG and low HDL TG < 500 mg/dL (5.6 mmol/L)	
Severe (> 1000 mg/dL)				
Familial chylomicronemia syndrome	AR Monogenic: defect in *LPL*, *APOC2*, *APOA5*, *LMF1*, *GPIHBP1*	hyperchylomicronemia	TG > 1000 mg/dL (11.2 mmol/L)	Dietary fat
Familial HTG	AD	Enlarged VLDL	TG > 500 mg/dL, (5.6 mmol/L) normal LDL-C and HDL-C levels	Can progress to severe HTG in the presence of secondary factors
Glycerol-3-phosphate dehydrogenase 1 deficiency	*AR* *GPD1* gene		Can have TG > 1000 mg/dL	transient infantile
Multifactorial CS	Multifactorial	Secondary factors*	TG > 1000 mg/dL (11.2 mmol/L)	

*AR* Autosomal Recessive, *AD* Autosomal Dominant, *HTG* Hypertriglyceridemia, *TG* Triglyceride, *LDL-C* Low Density Lipoprotein Cholesterol

### Etiology of mild-to-moderate HTG (< 1000 mg/dL)

1. **Polygenic HTG**: Common mild-to-moderate hypertriglyceridemia is typically multigenic, and results from the cumulative burden of common and rare variants in more than 30 genes, as quantified by genetic risk scores [7]. The interplay of genetic and secondary factors **(**Table 3**)** can trigger manifestations or exacerbation of HTG.

**Table 3:** Secondary Causes of Elevated Triglyceride Levels

Diet: highly processed nutrient dense food with high glycemic index or high fat content, high glycemic load, consumption of beverages containing sucrose or fructose, excess calorie intake
Endocrine disorders including uncontrolled type 1 and type 2 diabetes mellitus, obesity, metabolic syndrome, hypothyroidism, hypercortisolism, and lipodystrophies
Medications such as corticosteroids, oral estrogen, PEG-asparaginase, second generation antipsychotics, thiazides, isotretinoin, bile acid binding resins, non-cardio selective beta blockers, tamoxifen, cyclophosphamide, immunosuppressants, protease inhibitors, rosiglitazone, thiazide diuretics
Pregnancy
Renal disease such as nephrotic syndrome and renal failure
Liver disease including acute hepatitis, metabolic associated fatty liver disease
Excessive alcohol consumption
Chronic inflammatory conditions
2. **Primary HTG**: Specific conditions, such as   familial HTG, familial combined hyperlipidemia (FCHL), hypoalphalipoproteinemia, and familial dysbetalipoproteinaemia can cause primary HTG. However, it is important to note that, these conditions often requires  secondary factors or triggers, such as weight gain or IR, for HTG to fully manifest.

#### Familial hypertriglyceridemia (FHTG) is defined by an augmented production of exceptionally large triglyceride-enriched VLDL particles

Since these particles are larger, but not increased in number, apoB levels are generally normal in this scenario. Typically, individuals with this condition exhibit normal LDL-C and HDL-C levels and are not predisposed to premature CVD. FHTG typically presents with TG levels between 250 and 1000 mg/dL, normal- to-mildly elevated TC and low-to-normal LDL-C and HDL-C [41]. The risk of very severe HTG is heightened in the presence of predisposing conditions like type 2 diabetes (T2D).

#### Familial combined hyperlipidemia results from overproduction of apoB-100 relative to LDL and VLDL synthesis

Elevated TG is typically associated with elevated VLDL-C, leading to elevated apoB (apoB/LDL-C ratio > 1.0) and non-HDL-C levels. FCHL is an autosomal dominant disorder with variable penetrance, with a population prevalence of 2%–5%. At least 35 genes are implicated in FCHL development [42]. In individuals with a genetic predisposition, the manifestation of FCHL typically requires secondary conditions such as obesity, IR, or medication use such as oral contraceptives in females and retinoic acid for acne. The lipid phenotype varies within families and among individuals, often including isolated HTG (typically < 885 mg/dL or 2–10 mmol/L), concurrent elevations in LDL-C and TG, and increases in sdLDL. Individuals with FCHL face a heightened risk of premature CVD.

#### Dysbetalipoproteinemia is partly attributed to a mutation in the APOE gene (typically expressed as APOE3/E3 in the normal phenotype)

If the genotype is *APOE2/E2,* hepatocyte uptake of remnant lipoproteins is impaired. This mutation typically occurs in conjunction with a second genetic or acquired defect, leading to either overproduction of VLDL (as seen in FCHL) or reduced LDL receptor activity. This results in the accumulation of remnant particles, VLDL, and IDL. The lipid phenotype elevated  total cholesterol and TG levels, typically between 300 and 1000 mg/dL, with roughly equal levels [43]. Clinical dyslipidemia generally manifests in adulthood for men or before menopause for women. The dyslipidemia in this condition is diet-sensitive. Palmar xanthomas, orange lipid deposits in the palmar creases, are pathognomonic for this condition but are rarely seen in children.

#### Familial hypoalphalipoproteinemia (FHA) is a disorder characterized by elevated TG and low HDL-C, linked to premature CVD

Notably, genetic conditions leading to severely low HDL levels, such as Tangier's disease, apoA-I deficiency, and lecithin cholesterol acyl transferase deficiency, are also associated with varying degrees of HTG.

### Etiology of Severe HTG (≥ 1000 mg/dL)

1. **Familial Chylomicronemia Syndrome (FCS)**: Presents with early-onset severe HTG. FCS is an autosomal recessive disorder with with loss-of-function mutations in LPL pathway genes (LPL, LMF1, GPIHBP1, APOC2, and APOA5) [7].
  In FCS, HTG is typically caused by a significant accumulation of chylomicrons due to a pathogenic genetic variant that impairs LPL complex activity. Since chylomicrons do not traverse the vascular endothelial barrier because of their size, the risk of premature atherosclerosis is generally minimal, despite their markedly reduced level of HDL-C.
  FCS manifests early in infancy, childhood, or adolescence presenting with sustained fasting HTG of > 500 mg/dL,  although usually well over 1000 mg/dL, along with severe postprandial lipemia [44]. These patients experience recurrent pancreatitis and usually have a normal or low BMI. They can also present with eruptive xanthoma, lactescent blood, or lipemia retinalis. Apo B levels are low in these patients.
2. **Multifactorial chylomicronemia syndrome (MCS)**: MCS emerges later in childhood or adulthood. This occurs in patients with underlying genetic susceptibility for impaired TG metabolism (i.e., cluster of minor genetic variants) when exposed to environmental triggers such as obesity and IR, uncontrolled diabetes etc. Rarely this has been reported secondary to autoantibodies against components of the LPL complex [45]. TG levels fluctuate with intermittent hyperchylomicronemia, sometimes being normal or in the mild to moderate range. Elevated LDL-C levels may occur when TG levels improve. Unlike FCS, where chylomicrons accumulate, MCS involves accumulation of TRL remnants, with apo B concentrations usually above 100 mg/dL. Targeted next-generation sequencing for genes commonly associated with FCS is typically negative in MCS cases.
3. **Transient infantile HTG** can occur due to glycerol-3-phosphate dehydrogenase 1 deficiency from bi-allelic GPD1 gene mutations.

### Evaluation and Management of HTG

**Fig. 1:**
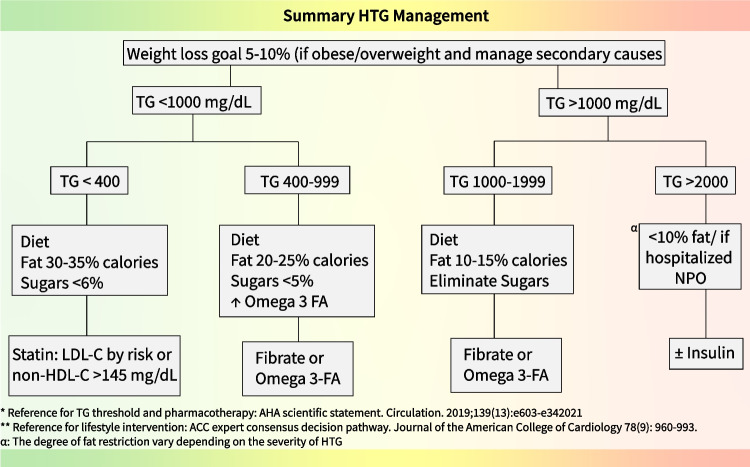
The figure outlines a comprehensive approach to managing elevated triglyceride levels expressed as milligrams/deciliter (mg/dL). The pathway emphasizes the percentage of total daily calories allowed to be used as fat and sugars under each triglyceride threshold levels. ^**α**^ Diet should avoid fat consumption or limit to <10% of total daily calories as fat. If hospitalized, NPO. Abbreviations: TG: triglyceride, LDL-C: Low density lipoprotein cholesterol, Non-HDL-C: non-high-density lipoprotein cholesterol, NPO: nothing per mouth, FA: fatty acids. Also, moderate to vigorous physical activity 1 h daily is recommended

Our management recommendations are based on a comprehensive review of multiple guidelines. We adopted the TG thresholds and pharmacotherapy recommendations from the AHA’s 2019 scientific statement for high risk pediatric patients since HTG is mostly associated with IR related conditions [16]. For lifestyle intervention strategies we adopted the ACC expert consensus pathway for managing ASCVD risk in patients with persistent HTG [46]. For severe and very severe HTG, we relied on the Endocrine Society's guidelines, along with other expert opinions and review articles [7, 47]. See Fig. 1.

### Mild to Moderate HTG (< 1000 mg/dL)

From a management perspective mild to moderate HTG is divided into TG < 400 mg/dL and TG 400–999 mg/dL.

#### TG < 400 mg/dL

Is usually due to increased hepatic synthesis of VLDL-C. VLDL generally consists of approximately 20% cholesterol and 80% TG. However, with increased hepatic production of VLDL an increase in total cholesterol levels occurs, making it a risk factor for premature ASCVD. It is commonly characterized by elevated TG, low HLD-C (usually below 40 mg/dL), and variable LDL-C levels. This combination is commonly called combined dyslipidemia (CD). CD is seen in about 30–60% of adolescents with obesity, namely combined dyslipidemia of obesity (CDO). While mild-to-moderate HTG is often associated with an unhealthy lifestyle and secondary factors, in some cases, it can result from cumulative effect of several minor gene variants, i.e., single nucleotide polymorphisms that can have consistent TG raising effects [48]. Overall CVD risk can be assessed using the risk assessment proposed by the AHA 2019 scientific statement [16]. A comprehensive family history of dyslipidemia and premature CVD is crucial.

#### TG 400–999 mg/dL

TG elevations in this range are reflective of a combination of increased VLDL synthesis and reduced clearance of TG from saturated or dysfunctional LPL activity. Hence further input of chylomicrons and VLDL lead to marked HTG and chylomicronemia. Routine genetic testing is not generally helpfulin cases of mild to moderate HTG.

### Evaluation Tools for mild-to- moderate HTG

**Fig. 2:**
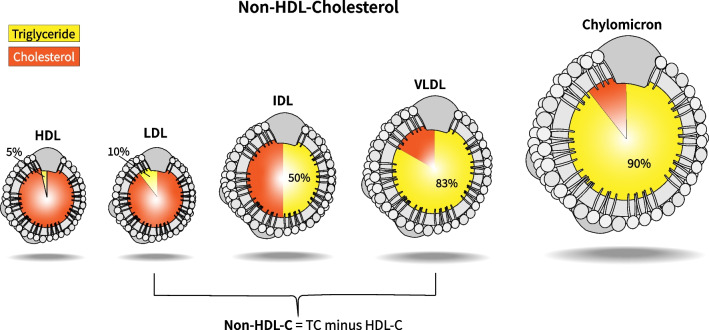
The figure illustrates the triglyceride (TG) and cholesterol concentrations within each lipoprotein. The triglyceride rich lipoproteins, i.e., chylomicrons, VLDL and IDL have varying cholesterol and TG concentrations. The triglycerides make up to 90% by weight of the chylomicron and 70–83% by weight of the VLDL. Non-HDL C is the aggregate total of all cholesterol contained in the atherogenic particles except that in the protective HDL. Non–HDL-C is a better predictor of cardiovascular risk than LDL-C in patients with hypertriglyceridemia

**Non-HDL cholesterol** [2, 14, 15], can guide management, particularly in non-fasting states and when TG concentrations are < 400 mg/dL. It represents the cholesterol content in atherogenic lipoprotein particles, including LDL-C, VLDL, and IDL- Fig. 2. Studies such as Pathobiological Determinants of Atherosclerosis in Youth Study (PDAY) and the Bogalusa Heart Study, underscore the importance in assessing CV risk [49, 50]. A cohort from the i3C Consortium (International Childhood Cardiovascular Cohorts) found that childhood non-HDL-C and LDL-C levels were associated with ASCVD events in midlife. Non-HDL-C was a superior predictor of adult ASCVD events compared to LDL-C, particularly among individuals with normal LDL-C but elevated non-HDL-C. This underscores its utility, especially as it does not require fasting [51].

#### Apolipoprotein B (apoB)

Apo B levels indicate the presence of total particle number of each apoB 100-containing lipoprotein particles, i.e. VLDL, IDL, LDL, and Lp(a) particle. In general, apo B levels are elevated in patients with CDO and IR where there is an increase in small dense LDL particles. In these cases, although the LDL-C levels might appearnormal or only slightly elevated, apo B would be discordantly high. In FCHL, apoB levels are typically persistently elevated, but in CDO the levels may decrease with loss and improvement of dyslipidemia. While measurement of apoB is currently not considered standard of care for children, there is increasing interest in its use to assess risk and to aid clinical decision-making.

#### TG/HDL-C ratio relevance

TG/HDL-C serves as a reliable predictor of CVD risk, indicating an atherogenic lipid profile and predisposition to CVD development. Numerous trials and studies support its effectiveness as a novel risk marker for predicting the CV risk. However, the lack of a universal threshold value poses challenges to its widespread clinical use as a predictive biomarker for CVD [52].

#### Key Strategies for Management: Lifestyle Changes, Addressing Secondary Factors, and Pharmacotherapy

It is important to note that reducing dietary fat and sugar is crucial for managing all types of hypertriglyceridemia, although the degree of restriction may vary depending on the degree of HTG. Dietary recommendation for TG < 400 mg/dL includes reducing simple sugar and high glycemic index foods, lowering fructose intake, and reducing total carbohydrate intake. Replace trans and saturated fats with monounsaturated fats and increase dietary omega-3 fatty acids [46]. It is recommended to reduce dietary fat to 20–25% of total calories and limit dietary sugars to less than 5%. See Fig. 1 (adapted from [46]).

Physical activity and exercise recommendations from NHLBI guidelines include 1 h/day of physical activity with vigorous intensity 3 days/week [2]. The AHA scientific statement recommends at least 5 h/week of moderate to vigorous activity [16]. TG can be reduced by 20–70% with 5–10% weight loss. Secondary causes of HTG should also be identified and treated.

### Pharmacotherapy for mild-to-moderate HTG

1. **TG < 400 mg/dL** (5.6 mmol/L): Management focuses on reducing global CVD risk. Because the rsik of cardiovascular disease is increased, treatment targets LDL-C first, then then non-HDL cholesterol if LDL-C is near the goal. A statin is the primary pharmacological treatment, with goal LDL-C below 130 mg/dL and non-HDL-C below 145 mg/dL. Statins can decrease plasma TG levels by up to 30%, depending on baseline concentration and dosage.
2. **TG ≥ 400 mg/dL** (5.6 mmol/L): The treatment target is fasting TG, typically using fibrates or omega-3 fatty acids ‘off- label’, since they do not have FDA approval in children. In patients with HTG, TG levels can increase up to four times after a meal, indicating impaired postprandial lipid clearance [18].
  Fibrates: Fibrates activate peroxisome proliferator-activated receptor alpha (PPARα), which helps regulate hepatic lipid metabolism. Fibrates lower hepatic TG production, inhibit VLDL-C production, and reduce peripheral lipolysis [53]. In children, their use is based on limited evidence [54–57]. These studies showed significant reductions in TG levels by up to 54% and increases in HDL-C by 17%. Long-term trials assessing the vascular or clinical response to fibrates in children are lacking. Although not FDA approved for youth less than 18 years of age, they are the primary treatment for reducing the risk of pancreatitis, potentially lowering TG by up to 40%.
  Omega-3 polyunsaturated fatty acids (FA), at doses of 4 g daily, can reduce TG concentrations by up to 30%, depending on baseline levels. There are three FDA-approved omega -3 FA formulations for adult use: Epanova (a mixture of Omega-3 FA in FFA form), Lovaza and Omtryg (eicosapentaenoic acid (EPA), docosahexaenoic acid (DHA) and docosapentaenoic acid), and Vascepa (icosapent ethyl or EPA ethyl esters). The two RCTs of omega-3 fish oil in adolescents did not show statistically insignificant reduction in TGs or change in LDL particle number or size [58, 59].

### Evaluation and Management of Severe HTG (TG ≥ 1000 mg/dL)

The primary objective of management is to prevent pancreatitis. The severe HTG is primarily caused by a substantial accumulation of chylomicrons stemming from impaired activity in the LPL-related TG clearing system. Chylomicron accumulation in the pancreatic capillary bed can result in abdominal pain, ischemia, and may progress to pancreatitis. In cases of very severe HTG, dietary fat restriction is crucial to reduce the chylomicron load. The management differs based on TG concentrations 1000–1999 mg/dL and > 2000 mg/dL [7, 47].

1. When the TG concentration is 1000–1999 mg/dL, dietary fat-restriction of 10–15% total daily calories is recommended [46]- this may equate to 15–20 g of fat intake per day. Other dietary interventions include eliminating added sugars, avoiding simple carbohydrates, reducing total carbohydrate intake, replacing trans and saturated fats with monounsaturated fats, and increasing dietary omega-3 fatty acids consumption. Additionally, it is essential to abstain from alcohol.
2. When TG concentrations exceed 2000 mg/dL, a ‘no fat’ diet or very low fat diet (<10% of total dietary calories) is very effective in lowering TG. If the patient is hospitalized, acutely restricting dietary intake (i.e. nothing per mouth or NPO) is also an effective strategy. Interventions like IV insulin infusion can lower TG levels by up to 40%. Combined with fasting, this approach can lead to an 80% reduction in TG within 24 [60]. Hospitalization is recommended for abdominal pain, pancreatitis, or multi-system involvement in very severe HTG. Interventions like with holding dietary intake and the administration of intravenous fluids and insulin (especially in patients with uncontrolled  diabetes and less so in others) can be helpful [60]. After addressing the risk for pancreatitis, management primarily relies on lifestyle interventions as above.
3. Plasmapheresis may be employed for the rapid mechanical removal of triglycerides in critically ill patients who are experiencing shock, severe pancreatitis, or are at risk of end organ failure.

### Pharmacotherapy for severe HTG

Fibrates are recommended as the primary treatment for lowering severe TG levels, even though they are not FDA approved for use in children. As TG levels decrease, LDL-C can increase due to enhanced conversion of VLDL to LDL. Fibrates showed no therapeutic benefit when the TG concentrations are ≥ 1770 mg/dL or > 20 mmol/L [7, 47]. It has been postulated that at these TG levels, chylomicrons coat the vascular lumen and prevent LPL from being available to hydrolyze the circulating TG.

Omega 3 FA have not shown significant TG reduction in children and adolescents and are generally not recommended due to their limited effectiveness and potential drawbacks. Since omega-3 FA themselves are a source of TG, using them in patients with severe HTG can be problematic. Each omega-3 FA capsule contains 1 gram of fat, so, if a patient’s diet is restricted to 15 grams of total fat intake per day and they take 4 omega-3 FA capsules, they are left with only 11 grams of fat from other sources. In cases where TG is >2000 mg/dL, omega-3 FA can actually worsen HTG.

### Emerging therapies

**Fig. 3:**
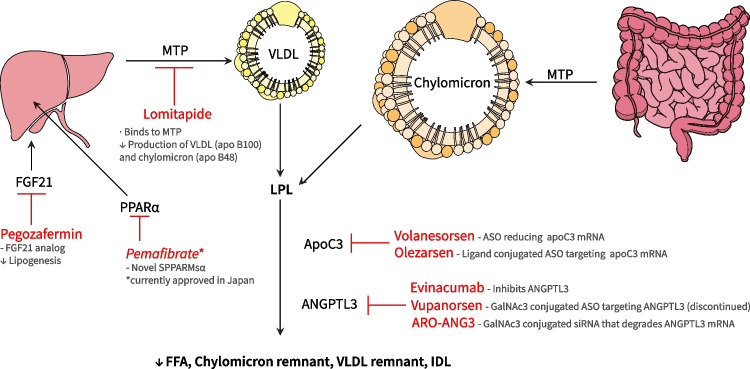
MTP: Microsomal triglyceride transfer protein, apoB: apolipoprotein B, VLDL- very-low density lipoprotein, TG: triglyceride, ASO: anti-sense oligonucleotide SPPAR-α: selective peroxisome proliferator-activated receptor alpha modulator, FGF21: Fibroblast growth factor 21, mRNA: messenger RNA, GalNac: N-acetylgalactosamine, ANGPTL3: Angiopoietin-like 3 proteins, LPL: lipoprotein lipase, VLDL- very-low density lipoprotein, FFA- free fatty acids,

While current treatments prove ineffective in very severe HTG, there are emerging novel therapies for lowering triglycerides focus on targeting specific pathways and mechanisms involved in lipid metabolism—Fig. 3.

#### Volanesorsen

Apolipoprotein C3 (apoC3) inhibits LPL. Volanesorsen, a second-generation antisense oligonucleotide (ASO), targets apoC3 mRNA to prevent translation and promote degradation, enhancing TG clearance through LPL-independent pathways. Clinical trials showed volanesorsen significantly reduced TG levels in adult patients with FCS [61, 62]. No RCTs exist on Volanesorsen in pediatrics, however a study involving a 13-year-old female with LPL deficiency demonstrated that it was able to reduce TG levels [63]. Safety concerns, particularly thrombocytopenia and injection-site reactions, have impacted its use. The European Medicines Agency conditionally authorized it for FCS [64], but the U.S. FDA rejected it due to these adverse effects.

#### Olezarsen

Olezarsen, or AKCEA-APOCIII-LRx, is a third generation ASO designed to lower apoC-III and TG levels. It employs a GalNac conjugate for enhanced tissue selectivity and improved first-pass clearance, potentially reducing the risk of thrombocytopenia and allowing for lower dosages compared to earlier drugs like Volanesorsen. Despite promising trials with significant decreases in TG, apoC-III, VLDL-C, non-HDL-C, and apoB levels in adults [65], there has not been a trial in pediatrics.

#### Lomitapide

Microsomal triglyceride transfer protein (MTP) is required for assembling apoB-containing lipoproteins in the liver (apoB-100) and intestine (apoB-48). Lomitapide functions by inhibiting MTP activity, reducing the production of apoB-100, and to a lesser extent, apoB-48. This MTP inhibitor may substantially lower plasma TG levels by inhibiting the formation and secretion of TRL remnants. Patients with Familial Abetalipoproteinemia, who have an MTP defect, have extremely low TC, with absent plasma VLDL, LDL-C, and apoB. There is an ongoing clinical trial in pediatric patients with homozygous familial hypercholesterolemia (HoFH) evaluating the efficacy and long-term safety of lomitapide (NCT04681170).

#### Evinacumab

Angiopoietin-like 3 (ANGPTL3), produced by hepatocytes, regulates lipid metabolism by inhibiting lipases, thereby affecting the lipolysis of VLDL and chylomicrons. Evinacumab is a monoclonal antibody that targets ANGPTL3 [66, 67]. In patients with severe HTG, evinacumab showed potential in reducing atherogenic lipids, despite mixed results on TG levels [68]. However, in a post-hoc analysis of three separate clinical trials, treatment with evinacumab in patients with hypercholesterolemia or HTG showed a reduction from baseline in TG-rich lipoprotein levels [69].

#### Vupanorsen

Vupanorsen, formerly known as IONIS-ANGPTL3-LRx and AKCEA-ANGPTL3-LRx, is an N-acetyl galactosamine-conjugated ASO that targets ANGPTL3 mRNA in the liver. Vupanorsen uses a GaINAc conjugate for selective liver uptake, reducing TG levels through LPL-independent pathways. Adult trials have showed positive results on reducing non-HDL-C, TG, and ANGPTL3 levels [70]. However, no pediatric trials have emerged.

#### ARO-ANG3

ARO-ANG3 is a synthetic siRNA molecule that targets hepatocytes to silence ANGPTL3 expression, reducing both ANGPTL3 and TG levels in the blood. Initial trials involving patients with hypercholesterolemia and heterozygous FH showed that TG levels were reduced by 25–43% in FH patients and by 29% in non-FH patients. This indicates that the treatment is effective, though the relationship between dose and response isn't clearly defined [71]. There are current ongoing trials further investigating (NCT04832971 and NCT05217667).

#### Mipomersen

Mipomersen, a second-generation ASO, targets apoB-100 production. Trials have shown significantly reduction in apoB, LDL-C, TC, TG, and Lp(a) [71]. However, it had significant side effects such as injection site reactions, flu-like symptoms, liver transaminase increases, and hepatic steatosis. Due to safety concerns, its use has been limited and was withdrawn by the FDA in 2019 due to hepatotoxicity risks.

Emerging pharmacotherapies for HTG include FGF21 analogs such as pegozafermin, and pemafibrate, a selective PPAR alpha modulator. LPL gene therapy has been investigated, with some studies indicating sustained improvements on pancreatitis, lipoprotein particles, and postprandial TG reduction for up to 12 weeks after injection. However, IM injection of a functional LPL gene (alipogene tiparvovec) into patient tissues showed no lasting effect after 12 weeks and the phase 4 clinical trial was discontinued by the end of 2017.

## Conclusion

Recent studies in adults highlight the significance of non-fasting TG levels as predictors of CVD risk, particularly in mild to moderate HTG. Newer therapies are emerging, particularly in FCS and MCS. Further research is needed to develop more effective treatment strategies, to reduce the pancreatitis risk particularly for severe cases of HTG.

## Data Availability

No datasets were generated or analysed during the current study.
